# Downregulation of GLYAT Facilitates Tumor Growth and Metastasis and Poor Clinical Outcomes Through the PI3K/AKT/Snail Pathway in Human Breast Cancer

**DOI:** 10.3389/fonc.2021.641399

**Published:** 2021-04-22

**Authors:** Xin Tian, Lina Wu, Min Jiang, Zhenyong Zhang, Rong Wu, Jianing Miao, Caigang Liu, Song Gao

**Affiliations:** ^1^ Department of Oncology, Shengjing Hospital of China Medical University, Shenyang, China; ^2^ Key Laboratory of Shengjing Hospital, China Medical University, Shenyang, China

**Keywords:** GLYAT, breast cancer, EMT, PI3K/AKT, clinicopathological features, prognosis

## Abstract

**Background:**

The Glycine N-acyltransferase (GLYAT) gene encodes a protein that catalyzes the transfer of acyl groups from acyl CoA to glycine, resulting in acyl glycine and coenzyme A. Aberrant GLYAT expression is associated with several malignant tumors, but its clinical importance in human breast cancer (BC), has yet to be fully addressed. This study aims to evaluate the clinical function of GLYAT in BC patients.

**Methods:**

GLYAT expression was determined by immune blot and immunohistochemistry in three BC cell lines and primary cancer tissues. The MDA-MB 231 cell line was used for GLYAT gene knockdown experiments while the MCF7 cell line for overexpression experiments. Colony formation experiments, soft agar experiments, and transwell assays were utilized for further inspection of cell proliferation and migration capabilities. Immunofluorescence and western blot were used to detect markers of the epithelial-mesenchymal transition (EMT) and changes in the PI3K/AKT/Snail pathway. The role of GLYAT in tumor growth and metastasis was also assessed in nude mice *in vivo*. Also, a correlation analysis was performed between clinicopathological features and GLYAT expression in BC patients.

**Results:**

GLYAT was decreased in human BC tissues and cell lines. Functional analysis showed that knockdown of GLYAT augmented BC cell proliferation *in vitro* and *in vivo*. However, this phenomenon was reversed when GLYAT was overexpressed in the transfected cells. Moreover, downregulation of GLYAT promoted the migratory properties of BC cells, likely through the activation of PI3K/AKT/Snail signaling, which subsequently induced the EMT. IHC analysis indicated that GLYAT was decreased in human BC tissues and lower GLYAT expression was correlated with histological grade, tumor TNM stage, Ki-67 status, and poorer survival in BC patients. Furthermore, lower GLYAT expression seemed as an independent risk factor related to poor prognosis in BC patients based on Cox regression analyses.

**Conclusion:**

Our findings demonstrate that downregulation of GLYAT expression in human breast cancer is correlated with EMT *via* the PI3K/AKT/Snail pathway and is also associated with histological grade, tumor TNM stage, Ki-67 status, and poor survival in breast cancer patients.

## Introduction

Breast cancer (BC) amongst women has been on the rise and it is fast becoming the most common malignancy in this population (1). Leading cancer statistics show that the incidence and mortality of breast cancer remain high worldwide (2, 3). Although surgery, chemoradiotherapy, endocrine therapy, and molecular targeted therapy have greatly improved the survival of BC patients, those with advanced BC are often left with very limited therapeutic options (4, 5). Many factors influence the prognosis and probability of responding to systemic therapies (e.g., TNM stage, ER, PR, and HER2 status), but clinically, we have observed that even patients with the same TNM stage, molecular typing, and other diagnostic markers still experience varied prognosis despite being given the same treatment modalities (4). While early diagnosis and initiation of treatment are crucial in improving survival rates of this debilitating disease, there is an urgent need for a deeper understanding of its molecular biology in order to aid in the development of more personalized and targeted treatment.

The EMT involves the dedifferentiation of epithelial cells into mesenchymal cells (6, 7). Cells which have undergone EMT demonstrate more malignant features that promote metastasis, treatment resistance, as well as relapse (8–10). The EMT participates in tumor invasion and metastasis through multiple pathways. Many studies have indicated the EMT is strongly linked to breast cancer development (11–13). The EMT is regulated by multiple factors, among which the primary signaling pathway is the PI3K/AKT pathway (14).

The glycine N-acyltransferase (GLYAT) gene was first discovered in the mitochondria of the bovine liver in 1953 and isolated in the human liver and kidneys in 1976 (15). It contains more than 23,000 base pairs over six exons and is situated chromosome 11 at position 11q12 (16). GLYAT catalyzes acyl group transfer from acyl CoA to glycine, resulting in acyl glycine and coenzyme A (acyl-CoA) (17). Several catabolic and anabolic reactions are catalyzed by acyl-CoA esters (18). Almost all catabolic reactions produce acyl-CoA, the product of which is an important source of oxidative phosphorylation and lipogenesis. Studies have shown that GLYAT expression is suppressed in human hepatocellular carcinomas and may be a critical molecule in the transition between the differentiation and carcinogenesis of liver cells (19). Nevertheless, literature is scarce surrounding GLYAT expression and its impact on human breast cancer.

The current investigation uncovered markedly suppressed GLYAT expression in breast cancer cells and tissues, which correlated with poorer prognosis and highly malignant clinicopathologic features in individuals with breast cancer. Interestingly, the EMT pathway appeared to be inhibited in the presence of GLYAT, working to decrease *in vivo* and *in vitro* breast cancer cell migration through alteration of the PI3K/ATK/Snail signaling pathway. In conclusion, our study is the first of its kind to implicate GLYAT to be a breast cancer anti-oncogene. These findings lay the foundation for future research on the biological behavior and targeted therapy of BC.

## Materials and Methods

### Datasets and GLYAT Expression Analysis

The public data used in this study were obtained from UALCAN dataset (http://ualcan.path.uab.edu/index.html) (20), GEPIA (http://gepia.cancer-pku.cn/index.html) (21), and the Human Protein Atlas dataset (http://www.proteinatlas.org) (22). For UALCAN data, we analyzed the heat map of GLYAT expression between normal and breast cancer samples; For GEPIA data, we scrutinized differently GLYAT expression level and survival data in breast cancer. The relationship between patient survival and GLYAT levels were also analyzed using information available from the Human Protein Atlas data.

### Cell Lines

Human BC cell lines MDA-MB-231, MCF-7, and SKRB-3 were supplied by The Stem Cell Bank, Chinese Academy of Sciences (Shanghai, China). Cells were maintained at 37°C in a humidified cubicle containing 5% CO_2_ and 10% fetal bovine serum (FBS) in DMEM (all from Gibco, Carlsbad, CA, USA).

### Plasmid Formation of GLYAT Knockdown and Overexpression in Breast Cancer Cells

The pGPU6/mCherry/Puro-shRNA-GLYAT plasmid was purchased from GenePharma Company (Shanghai, China). The pOGP-T2A-CKNeo-GLYAT overexpression plasmid was purchased from JTS Scientific Company (Wuhan, China). The target sequences of GLYAT were indicated in **Supplementary Table 1**. Lipofectamine 3000 (Thermo Fisher Scientific, US) was used to transfect plasmids into cells as reported by the manufacturer’s instructions. Cells transfected with GPU6/mCherry/Puro-shNC or pOGP-T2A-CKNeo-NC were used as controls. Transfected cells were screened using 3 ug/mL puromycin or 400 ug/mL G418 for 2 weeks. Stable cells were cultured in complete medium with 0.25 ug/mL puromycin or 100 ug/mL G418 (Beyotime, Nanjing, China). Positive clones were then selected and amplified for further analyses.

### Cell Proliferation Assay

For colony forming experiments, six-well plates were used to house stable GLYAT-shRNA and NC cells. The cells were maintained at a concentration of 500 cells/well for 12 days in DMEM supplemented with 10% FBS, with media changed once every three days. Cells were then methanol-fixed and treated with crystal violet (Sigma, St Louis, MO, USA) prior to manual counting and photographing of the visible colonies. For soft agar experiments, Cells are harvested and pipetted well to become single-cell suspension in complete culture media in 1x 106/ml. A mixture of 0.9 ml 4% soft-agar (Sigma) with 4.1 ml pre-warmed 10% FBS DMEM was added into a 60-mm culture dish to make the bottom layer. The top layer contained 3 x 104 cells in 3 ml of 10% FBS DMEM and 0.36% agar. The soft-agar colony dish was marked and placed at a 37°C incubator for 3 weeks. cells were harvested and fully mixed to form a single-cell suspension in media of 1 x 10^6^/ml. The upper layer included 3 x 10^4^ cells, 3 ml of 10% fetal bovine serum, DMEM, combined with 0.36% agar (Sigma), while the bottom layer contained 0.9 ml 4% soft-agar, 4.1 ml pre-heated 10% fetal bovine serum, and DMEM in a 60-mm culture dish. Labeled colony dishes were placed in an incubator at 37°C for 3 weeks. Ten fields of each group were selected and the colony size was measured using Image J software (vision 1.53 National Institutes of Health, USA). All experiments were performed in triplicate and repeated at least three times.

### Transwell Migration Assay

Costar chambers containing 8 μm pore inserts were used (Millipore-Sigma, Danvers, MA, USA). The top chamber was used to house suspended cells transfected with GLYAT KD, GLYAT OE, or NC in 200 μl of serum-free medium, while media with 20% FBS was applied to the bottom chamber. The upper chamber was removed after 36 hours of incubation, with the bottom cartridge fixed with methanol and DAPI-stained to determine the migrated number of cells using a microscope to visualize the cells across five randomly selected fields (Olympus, Tokyo, Japan). Cell numbers were quantified manually.

### Western Blot Analysis

The proteins in BC cells were first extracted and separated. Skimmed milk (5%) was used to block endogenous reactions. Membranes were left to incubate with antibodies of GLYAT (1:1000, Abcam, Cambridge, MA, USA), E-cadherin, vimentin, N-cadherin, fibronectin (1:1000 dilution respectively, Cell Signaling Technology, Boston, MA, USA), p-AKT Ser473, AKT (1:1000 and 1:5000 dilution, Signalway Antibody, Baltimore, MD, USA), PI3K p58 and SNAI1 (1:10000 and 1:1000 dilution, Proteintech, Rosemont, IL, USA) in agreement with the protocols set by the manufacturer. Anti-β-actin (1:10000 dilution, Proteintech, Rosemont, IL, USA) was used as the internal control. After three rinses with TBS-T for 5 minutes, the membranes were incubated with the corresponding secondary antibody and observed with an ECL Plus kit. The relative levels of individual target proteins to the control were determined by the densitometric analysis using the ImageJ software.

### Immunofluorescence Staining

Cells were subjected into 8 well chamber slides (Millipore-Sigma, Danvers, MA, USA) and for 20 hours incubation then rinsed with PBS containing 10% FBS. Pre-cooled methanol was used to fix the cells prior to further incubation with 0.2% Triton. BSA (5%) was used to block cells. The cells were next incubated overnight with primary antibodies E-cadherin, vimentin (1:200 dilution respectively) before being washed and re-incubated with fluorescein-conjugated secondary antibodies (1:500 ZSGB-Bio, Beijing China) for one hour. Then, DAPI and a fluorescence microscope (Olympus, Tokyo, Japan) were used for visualization and intensity analysis. For dewaxed sections, the process was similar like above, and primary antibodies dilution of E-cadherin, vimentin and p-AKT Ser473 were 1:100 respectively.

### *In Vivo* Metastasis Assay

The HFK BIOSCIENCE CO., LTD (Beijing, China) provided 5 weeks old BALB/c (nu/nu) female nude mice (18–20 g). These animals were divided into four cohorts with five mice each. Food and drinking water were provided every day. All mice received subcutaneous injections into their left breast fat pads with 100 microliters of PBS containing 2.0 × 10^6^ MCF7 cells with or without GLYAT OE and MDA-MB-231 cells with or without GLYAT KD. At time of MCF7 cells injection, E2 pellets (60-day release, 1.5 mg/pellet; Innovative Research of America, Sarasota, Florida USA) was implanted subcutaneously at the mammary fat pad. Four days measurements and volume calculation ((mm^3^) =length × width^2^/2) of the tumors were performed. The mice were sacrificed and underwent tumor removal after 24 days. The tumors were processed with 4% paraformaldehyde solution for immunofluorescence staining analysis.

### Patients and Samples

Two independent breast cancer patient cohorts from the Shengjing Hospital of China Medical University were collected for this investigation. The first cohort consisted of 21 breast cancer patients (seven for Luminal A/B, seven for HER2 positive, and seven for TNBC) and 20 normal controls. Samples from the cancer patients and normal controls were analyzed for GLYAT expression. The second cohort comprised of 310 chemoradiotherapy-naïve breast cancer patients who had distinctive pathological diagnoses and possessed complete follow-up data from 2006 to 2008. Additional follow-up of these patients was carried out until September 2012.

### Immunohistochemistry

The dewaxed sections were retrieved with Tris/EDTA (pH9.0) and quenched with 0.3% H_2_O_2_. Sections then underwent an overnight incubation with GLYAT primary antibody (1:100, Abcam, Cambridge, MA, USA). PBS was used to rinse the samples prior to repeat incubation with secondary biotinylated antibodies at room temperature for 45 minutes. Coloration was visualized with 3,3 ‘-diaminobenzidine tetrachoric acid (Sigma-Aldrich), Vector hematoxylin QS (Vector Laboratories) was reverse-stained, Zeiss Mirax MidiSlide scanner was analyzed, and image collection was performed with 3 CCD color cameras and Panoramic Audience (3DHISTECH, Budapest Hungary). H-score was calculated as the percentage of positive tumor cells multiplied by the intensity of staining (0, no staining; 1, weak; 2, mild to moderate; 3, strong staining) and the overall H-score ranged from 0 to 300. An H-score of more than 100 was set as cut-off value to define high/low level for GLYAT expression.

### Statistical Analysis

Data were analyzed using SPSS version 25.0 (SPSS, Inc., Chicago, IL, USA) and expressed as means ± SD. The Kaplan-Meier method was used to estimate survival curves. Cox regression model was applied to carry out univariate and multivariate statistical analysis. Comparison between two groups was assessed by Chi-square. *P* value of less than 0.05 was considered statistically significant.

## Results

### Characterization of GLYAT Expression Profiling in Human BC

**Figure 1:**
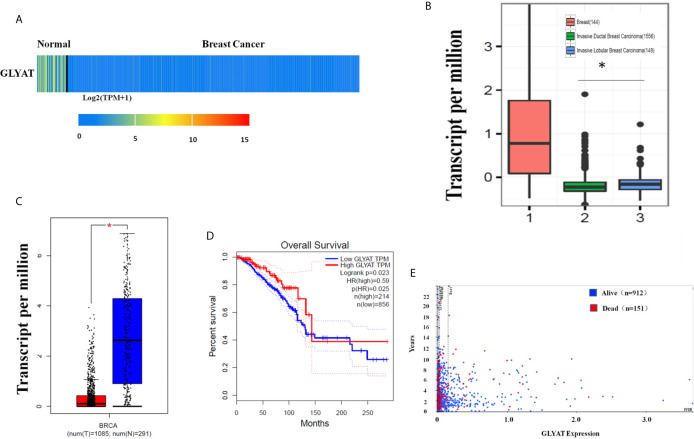
Characterization of GLYAT expression profiling in human breast cancer. **(A)** Heatmaps showing the clustering patterns of differentially expressed GLYAT between normal and breast cancer specimens. **(B)** Box plot analysis of the GLYAT mRNA levels in IDBC, ILBC and normal breast tissue samples. **(C)** Box plot analysis of the GLYAT mRNA levels in BC and NC tissues. T, tumor; N, normal. **(D, E)** Kaplan-Meier plots of OS in BC patients with different levels of GLYAT. *p < 0.05.

The heat map and box plot analysis of the UALCAN and GEPIA dataset showed lower expression of GLYAT protein and mRNA in BC tissues in contrast to healthy samples (**Figures 1A, C**). However, the expression of GLYAT was not statistically significant change in other cancer types, besides BRCA, LIHC and CHOL (**Supplementary Figure 1**), which indicated that GLYAT may have specific function in breast cancer. Additionally, analysis of data published in another study in *Nature* (23) which compared mRNA expression levels of GLYAT in normal breast tissues with invasive ductal and invasive lobular carcinoma revealed that two breast carcinoma subtypes had lower *GLYAT* mRNA levels (**Figure 1B**). A Kaplan-Meier analysis from GEPIA dataset and a scatter plot from the Human Protein Atlas dataset suggested that patients with low GLYAT expression in breast cancer tissues had worse overall survival (OS) (**Figures 1D, E**), further highlighting the suppressive function of GLYAT in BC progression.

### GLYAT Suppresses BC Cell Proliferation and Metastasis

**Figure 2:**
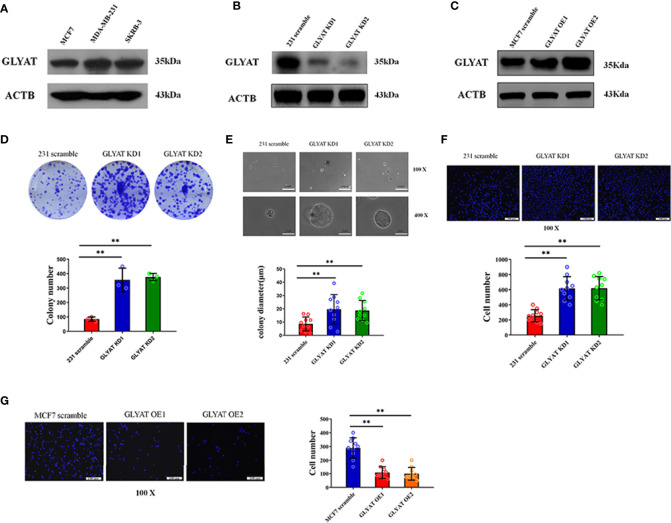
GLYAT suppresses BC cell proliferation and metastasis. **(A)** GLYAT protein level was assessed in different BC cell lines. **(B)** MDA-MB-231 cells were transfected with GLYAT KD plasmid and a scrambled plasmid as control. The suppression of GLYAT in MDA-MB-231 cells was confirmed at the protein level. **(C)** MCF-7 cells were transfected with GLYAT OE plasmid and a scrambled plasmid as control. The over expression of GLYAT in MCF-7 cells was confirmed at the protein level. **(D, E)** GLYAT KD in MDA-MB-231 cells significantly increased colony numbers and size compared with the scramble cells. **(F)** Transwell assay revealed that the migration ability of MDA-MB-231 cells was significantly increased following transfection with GLYAT KD1 and KD2 compared with the scramble control. **(G)** Transwell assay revealed that the migratory ability was significantly decreased in MCF-7 cells GLYAT OE compared with scramble control. **p < 0.01. Scale bars for **(E)** are 100μm and 25μm, and for **(F, G)** are 100μm.

Three typical breast cancer cell lines were scrutinized for GLYAT expression. These cell lines included SK-BR3 (Her-2 positive subtype), MDA-MB-231 (triple negative subtype) and MCF-7 (luminal A subtype). Western blotting showed that the MDA-MB-231 cell line had the highest GLYAT expression, while the MCF-7 cell line had the lowest amount (**Figure 2A**). Therefore, these two cell lines above were selected for further experiments.

*In vitro* assays were carried out in MDA-MB-231 cells with GLYAT knockdown (KD) and overexpression (OE) in MCF-7 cells to determine the impact of GLYAT expression on breast cancer behavior (**Figures 2B, C**). The tablet colony formation and soft agar colony formation experiments revealed that GLYAT KD in MDA-MB-231 cells significantly increased colony numbers and size in contrast to control cell lines (**Figures 2D, E**). Transwell migration assay revealed artificially altered MDA-MB-231 cells with GLYAT KD also had stronger migratory abilities in contrast to the control cells (**Figure 2F**). In line with these results, the converse was seen in MCF-7 cells with OE GLYAT (**Figure 2G**). These results indicate that GLYAT may hinder the proliferative and metastatic abilities of breast cancer cells.

### GLYAT Suppresses the EMT Phenotype in BC Cells

**Figure 3:**
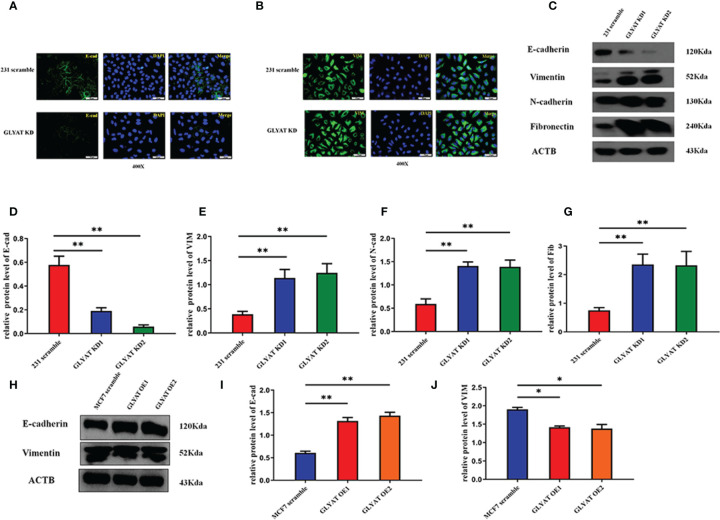
GLYAT suppress EMT phenotype in BC cells. **(A, B)** Immunofluorescence assay showed that E-cadherin expression was decreased and the vimentin expression was increased after the treatment of GLYAT KD. **(C–G)** Protein levels of E-cadherin was reduced, whereas the levels of Vimentin, N-cadherin, and Fibronectin were increased in MDA-MB-231 cells following GLYAT inhibition. **(H–J)** Protein levels of E-cadherin was increased whereas the expression of Vimentin was reduced in GLYAT OE MCF-7 cells. *p < 0.05, **p < 0.01. Scale bars are 25μm.

Given the prominent role of EMT in tumor spread, we postulated that GLYAT may be involved in the EMT process. *In vitro* assays were performed in MDA-MB-231 with GLYAT KD and in MCF with GLYAT OE. Both vimentin and E-cadherin were analyzed for their nucleic location *via* immunofluorescence assays. In GLYAT KD cells, we found an increase in vimentin but a decrease in E-cadherin expression (**Figures 3A, B**). Western blotting of EMT-related proteins was consistent with the immunofluorescence results, showing a reduction in E-cadherin, but increase in levels of vimentin, N-cadherin, and fibronectin in MDA-MB-231 GLYAT KD cells (**Figures 3C–G**). On the contrary, MCF-7 GLYAT OE cells demonstrated raised E-cadherin protein expression and lowered vimentin levels (**Figures 3H–J**). These results suggest that EMT formation could be suppressed by GLYAT.

### GLYAT Suppresses the EMT of BC Cells *via* the PI3K/Akt/Snail Pathway

**Figure 4:**
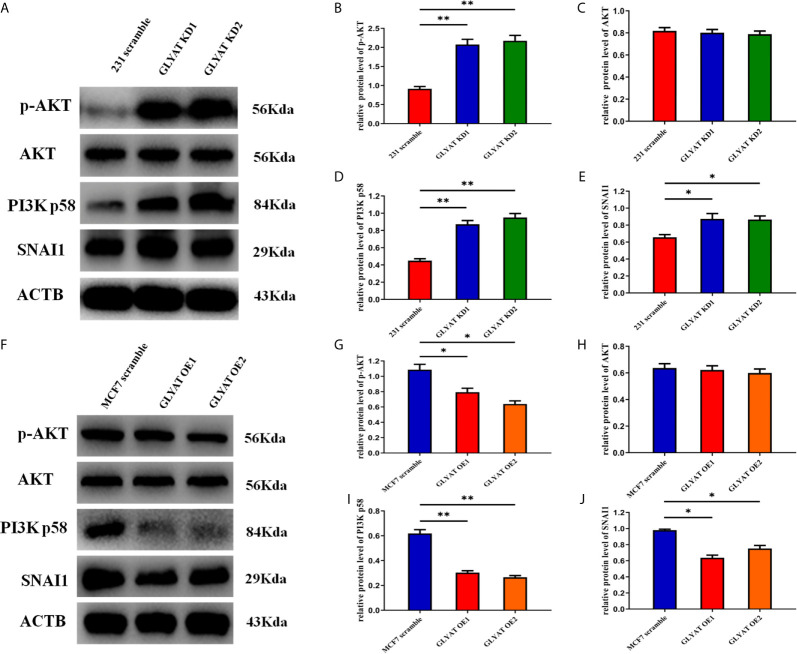
GLYAT regulated breast cancer cells EMT progression through PI3K/Akt signaling Pathway. **(A–E)** Protein levels of p-AKT,AKT, PI3K and SNAI1 were significantly activated in GLYAT KD MDA-MB-231 cells. **(F–J)** Protein levels of p-AKT,AKT, p-PI3K and SNAI1 were significantly reduced in GLYAT over expressed OE MF-7 cells. *p < 0.05, **p < 0.01, and p < 0.05 was considered to be statistically significant.

EMT process is highly regulated by the PI3k/Akt/Snail pathway. We therefore hypothesized that GLYAT might suppress breast cancer metastasis by modulation of this pathway. Western blot assays indicated that p-AKT, AKT, PI3K p58, and SNAI1 were significantly activated in cells with GLYAT KD (**Figures 4A–E**). OE cells on the other hand, demonstrated markedly suppressed levels of p-AKT, AKT, PI3K p58, and SNAI1 in contrast to control cells (**Figures 4F–J**). Our results indicate the involvement of the PI3K/Akt/Snail signaling pathway in GLYAT-mediated EMT suppression in BC cells.

### GLYAT Suppresses *In Vivo* BC EMT, Proliferation and Metastasis

**Figure 5:**
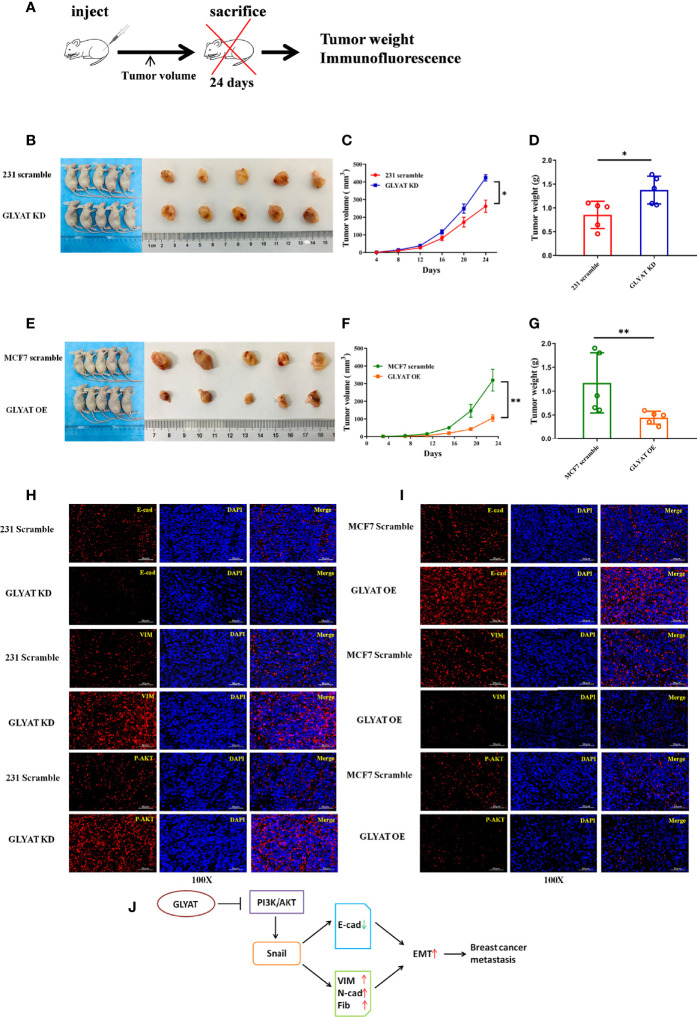
GLYAT suppress breast cancer proliferation, metastasis and EMT *in vivo*. **(A)** Schematic diagram of the metastasis model in mice. **(B–D)** The mice injected with stable GLYAT KD MDA-MB-231 cells had markedly bigger and heavier tumors. **(E–G)** The mice injected with stable GLYAT OE MCF-7 cells had markedly smaller and lighter tumors. **(H)** Immunofluorescence assay of serial sections mouse tumor tissues revealed that GLYAT KD MDA-MB-231 cells significantly decreased the expression of E-cadherin whereas increased the expression of Vimentin and p-AKT. **(I)** Immunofluorescence assay of serial sections mouse tumor tissues revealed that, in GLYAT OE MCF-7 cells, the expression of E-cadherin was increased whereas Vimentin and p-AKT were decreased. **(J)** The schematic diagram of the role of GLYAT in BC. *p < 0.05, **p < 0.01. Scale bars are 50μm.

*In vivo* experiments were performed on nude mice that were received subcutaneous injection with either stable MDA-MB-231 GLYAT KD or MCF-7 GLYAT OE cells (**Figure 5A**). The mice injected with stable GLYAT KD cells developed bigger and heavier tumors (**Figures 5B–D**). In contrast, the mice injected with stable GLYAT OE MCF-7 cells had markedly smaller and lighter tumors (**Figures 5E–G**). Then immunofluorescence assays for E-cadherin, vimentin, and p-AKT were performed using serial sections of mouse tumor tissues. These assays revealed that GLYAT silencing in MDA-MB-231 cells significantly decreased E-cadherin levels, but increased vimentin and p-AKT levels (**Figure 5H**). In contrast, in GLYAT OE MCF-7 cells, E-cadherin was increased, whereas vimentin and p-AKT were decreased (**Figure 5I**).

### Lower GLYAT Expression Is Correlated With Poorer Prognosis and Malignant Clinicopathological Features in Human Breast Cancer Tissues

**Figure 6:**
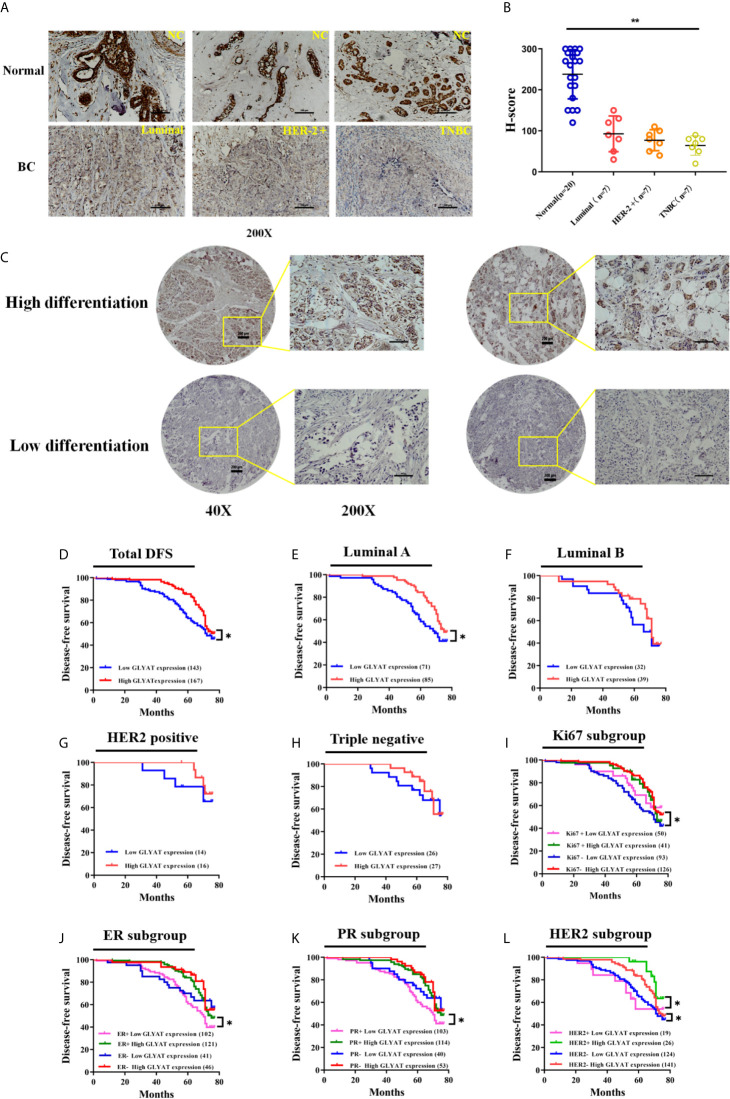
GLYAT is less expressed in BC tissues and correlated with worse survival. **(A, B)** Immunohistochemical staining showed that GLYAT was downregulated in BC tissues compared with NC. **(C)** Immunohistochemical staining showed that the lower differentiation of the breast cancer, the less expressed of GLYAT in breast cancer tissues. **(D)** Kaplan-Meier analysis of DFS in BC patients with different levels of GLYAT. **(E–H)** Kaplan-Meier analysis of DFS in luminal A, luminal B, HER2 positive and triple negative BC patients with different levels of GLYAT. **(I–L)** Kaplan-Meier analysis for DFS by Ki67, ER, PR, HER2 status in BC patients with different levels of GLYAT. *p < 0.05, **p < 0.01. Scale bars for **(A)** are 200μm, and for **(B)** are 50μm and 200μm.

Immunohistochemical experiments found that breast cancer tissues possessed markedly decreased GLYAT expression in contrast to healthy breast tissue (**Figures 6A, B**). Additionally, GLYAT expression in BC tissues correlated positively with the degree of pathological differentiation. (**Figure 6C**). Based on these results, we conclude GLYAT may act as an anti-oncogene in BC.

We then studied the correlation between clinicopathological features and GLYAT expression in 310 patients who were recruited for this investigation. **Table 1** demonstrates their characteristics. All individuals were women between 29 to 78 years old. No one had a previous history of malignancy and were chemoradiotherapy-naïve. The patients were grouped based on their GLYAT levels-low (n=143) or high (n=167) expression groups. We found GLYAT expression correlated with TNM stage, histological grade, and Ki-67 status (**Table 1**; all *P*<0.05). Additionally, we did not uncover any relationship between GLYAT level and menopausal status, age, receptor status (HER2, ER, PR), tumor size, node status, pathologic or molecular subtype (**Table 1**; all *P*>0.05). These findings suggest GLYAT is associated with malignant clinicopathological characters of BC.

**Table 1:** Correlation between GLYAT expression level and clinicopathological features in patients with breast cancer.

Clinical characteristics	No. (*n = *310)	GLYAT		*P* value
		Low (143)	High (167)	
**Age (years)**				
<50	139	59	80	0.241
≥50	171	84	87	
**Menopausal status**				
Premenopausal	165	73	92	0.477
Postmenopausal	145	70	75	
**Tumor Size (cm)**				
≤2	193	93	100	0.351
>2	117	50	67	
**Nodes status**				
Positive	102	41	61	0.142
Negative	208	102	106	
**TNM stage**				
I	128	40	68	0.033
II	133	64	69	
III	49	39	30	
**Pathologic type**				
Invasive ductal carcinoma	202	97	105	0.361
Others	108	46	62	
**Histological grade**				
I	51	16	35	0.040
II	201	95	106	
III	58	32	26	
**ER status**				
Positive	223	102	121	0.826
Negative	87	41	46	
**PR status**				
Positive	217	103	114	0.471
Negative	93	40	53	
**Her-2 status**				
Positive	45	19	26	0.570
Negative	265	124	141	
**Ki-67 status**				
High	91	50	41	0.045
Low	219	93	126	
**Molecular subtype**				
Luminal A	156	71	85	0.971
Luminal B	71	32	39	
HER2 Positive	30	14	16	
Triple Negative	53	26	27	

GLYAT, Glycine N-acyltransferase; TNM, tumor-node-metastasis.

BC patients were analyzed for their GLYAT status and prognosis *via* Kaplan-Meier survival analysis as well as log-rank tests after being followed up for an average of 61.75 months (range, 9–77 months). We found that those with decreased GLYAT levels experienced poorer disease-free survival (DFS) (**Figure 6D**; P=0.012). GLYAT expression was a significant indicator of DFS rates in breast cancer patients based on univariate Cox regression analysis, demonstrating a hazard ratio (HR) of 1.565 [P<0.05; 95% confidence interval (CI) 1.098-2.232] (**Table 2**). Further multivariate analysis revealed that GLYAT expression was significantly related to DFS (HR, 0.145; 95% CI, 0.031-0.690; P=0.015) (**Table 2**) and represented an independent risk factor of prognosis for BC.

**Table 2:** Univariate and multivariate analyses of clinicopathological risk factors for disease-free survival among breast cancer patients.

	DFS			
Variables	Univariate analysis		Multivariate analysis	
	HR (95% CI)	P value	HR (95% CI)	P value
Age	1.109 (0.778-1.580)	0.567	1.779 (0.995-3.182)	0.052
Menopausal status	0.835 (0.586-1.191)	0.321	0.562 (0.313-1.009)	0.053
T stage	1.139 (0.784-1.653)	0.044	0.457 (0.245-0.851)	0.014
N stage	0.951 (0.655-1.381)	0.023	0.466 (0.235-0.927)	0.029
TNM stage	0.630 (0.375-1.058)	0.043	0.432 (0.225-0.741)	0.004
Pathological type	0.914 (0.633-1.319)	0.630	0.937 (0.635-1.383)	0.744
Histological grade	1.035 (0.580-1.846)	0.908	0.878 (0.403-1.914)	0.743
ER status	0.726 (0.480-1.097)	0.128	0.510 (0.215-1.210)	0.127
PR status	0.737 (0.496-1.095)	0.131	0.788 (0.422-1.469)	0.453
HER-2 status	1.181(0.618-2.257)	0.615	1.986 (0.689-5.726)	0.204
Ki-67 status	0.864 (0.574-1.300)	0.483	1.408 (0.621-3.192)	0.412
Molecular subtype	1.677 (0.925-3.041)	0.089	2.910 (0.696-4.177)	0.143
GLYAT status	1.565 (1.098-2.232)	0.013	1.475 (0.991-2.197)	0.046

Patients were then divided into five subgroups based on their molecular subtype, Ki67, ER, PR and HER2 status in order to investigate the effect of GLYAT on prognosis.

In the subgroup analyses by molecular subtype, patients were grouped into triple negative or HER2 positive, Luminal A, or Luminal B subgroup. We found that GLYAT had a negative impact on DFS in all groups, especially in the Luminal A subgroup, where there was a statistically significant difference (*P*=0.014, **Figures 6E–H**).

In the subgroup analyses based on Ki67, those with low GLYAT levels were found to have shorter DFS in comparison to those with high GLYAT, regardless of whether they had high or low Ki67. This difference was statistically significant in those with low Ki67 (*P*=0.014, **Figure 6I**). Similarly, those with low GLYAT levels also experienced shorter DFS in contrast to those with high GLYAT, irrespective of ER+ or ER- status. There was a significantly statistical difference in those of ER+ status (*P*=0.011, **Figure 6J**). The same pattern of findings also applied to subgroup analyses based on PR. We also observed a statistically significant difference in the subgroup of PR+ patients (*P*=0.011, **Figure 6K**). Likewise, HER2 status did not change the impact of low GLYAT on shorter DFS, with statistically significant changes observed in both HER+ or HER- status groups (*P*=0.046 and *P*=0.045, **Figure 6L**).

## Discussion

Advanced breast cancer is a condition that carries high morbidity and mortality, despite the existence of several diagnostic markers and treatment strategies (24). Therefore, we sought to determine novel molecular components involved in the biology of BC that may be useful in the management of this condition.

A potential candidate is the GLYAT protein, which is involved in glycine conjugation of xenobiotics such as benzoic acid, and plays a role in many anabolic and catabolic reactions (25). In cancerous cells, GLYAT functions as a key metabolite that inhibits glycine uptake or biosynthesis, impairing growth of these cells likely through inhibition of nucleic acid synthesis (26). Previous reports have highlighted the role of this molecule in musculoskeletal growth and development as well as in hepatocellular carcinoma progression (19). Nevertheless, there has been no study on its oncological effect in breast cancer.

Our series of experiments found that human breast cancer cells and tissues contained remarkably suppressed levels of GLYAT. We further uncovered that lower GLYAT level was associated with more malignant clinicopathological characteristics, including higher TNM stage, histological grade, and Ki-67 status. DFS of those with lower GLYAT levels were also shorter. Our findings are consistent with information available in public databases. As breast cancer is related to hormone and has different molecular types, we further divided patients into five separate subgroups based on molecular subtype, Ki67 status, ER status, PR status, and HER2 status to analyze the correlation between GLYAT expression and prognosis of breast cancer. We found that breast cancer patients with low expression levels of GLYAT had poorer DFS regardless of the molecular subtype, Ki67 status, ER status, PR status, and HER2 status. Especially in the Luminal A subgroup, the Ki67 low status subgroup, the ER+ subgroup, the PR+ subgroup, and both the HER+ and HER- subgroups, there was a statistically significant difference (all P<0.05). We postulate that the lack of statistical significance for other subgroups, such as the Ki67 high status subgroup, the ER- subgroup, and the PR- subgroup, can be attributed to small sample sizes or an inadequate follow-up time. However, perhaps more importantly, ER and PR status are intricately linked to breast cancer initiation, development and prognosis. GLYAT may act in combination with ER or PR status to impact breast cancer prognosis. Additional experiments are needed to clarify this question in further research.

We further discovered GLYAT to be an independent prognostic factor for BC. These findings indicated that GLYAT acts as antioncogene and is associated with malignant clinicopathological features that may enhance breast cancer metastasis and progression. Our findings based on DFS alone are suggestive of the potential of this molecule to prognosticate breast cancer. Our findings are consistent with bioinformatics analyses and the data reported by Matsuo M et al., who indicated that GLYAT was suppressed in human hepatocellular carcinomas (19). Given that our study is the first to analyze the correlation between GLYAT and breast cancer, future investigations and follow up investigations regarding OS information (not available in our study due to short follow-up time) are still required.

We further demonstrated that GLYAT regulates breast cancer migration and invasion *via* EMT modulation. EMT is reduced *via* alteration of the PI3K/ATK/Snail signaling pathway both *in vitro* and *in vivo*. The EMT process participate in cancer metastasis (27). Numerous studies reported that EMT is regulated by several signaling pathways, for example Notch-, Wnt-, PI3K/Akt- and NF-κB-dependent pathways (28–30). Previous studies have confirmed that the EMT enhances tumor cell mobility and invasiveness, thus, contributing to the development of chemotherapy resistance and metastasis (31, 32). Additionally, breast cancer recurrence and prognosis have been reported to be affected strongly by EMT (13). We believe there may be other mechanisms in addition to the PI3K/AKT/Snail pathway by which GLYAT promotes metastasis that need to be further explored. One example by Ren et al. using a Drosophila model demonstrated GLYAT to be a critical modulator of the c-Jun N-terminal kinase (JNK) signaling pathway (33). GLYAT downregulation suppresses JNK-dependent ROS activation, which is a key facilitator of several important biological functions.

Besides the impact on metastasis, the proliferation ability of BC cells was increased after inhibiting GLYAT both *in vitro* and *in vivo*, further highlighting the tumor suppressed role of GLYAT. Our research is the only study to illustrate the role of GLYAT in BC patients, revealing that down-regulated GLYAT induces EMT and tumor metastasis *via* PI3K/AKT/Snail signaling.

## Conclusion

Collectively, our data revealed that GLYAT downregulated in human BC cells and tissues, and lower GLYAT expression was related to poor clinical outcomes. GLYAT suppresses BC cell proliferation and migration through EMT induction *via* the PI3K/AKT/Snail pathway *in vitro* and *in vivo*. Our study puts forth GLYAT as a novel biomarker in BC and may also represent a therapeutic target for BC treatment.

## Data Availability Statement

The original contributions presented in the study are included in the article/**Supplementary Material**. Further inquiries can be directed to the corresponding author.

## Ethics Statement

The studies involving human participants were reviewed and approved by the ethics committee of Shengjing Hospital of China Medical University (NO.2018PS234K). The patients/participants provided their written informed consent to participate in this study. The animal study was reviewed and approved by the ethics committee of Shengjing Hospital of China Medical University (NO.2018PS234K). Written informed consent was obtained from the individual(s) for the publication of any potentially identifiable images or data included in this article.

## Author Contributions

SG conceived and designed the experiments as well as contributed to the writing of the manuscript. XT, NW, MJ, YZ, NM, and RW performed the experiments. GL provided the tumor tissue microarray and brief guidance about conception at the beginning of the study. XT performed clinical analysis and helped with drawing the figures. SG revised the paper. All authors contributed to the article and approved the submitted version.

## Funding

The study was funded by the Doctoral start-up foundation of Liaoning Province (No. 20180540024), and 345 Talent Project, Shengjing Hospital of China Medical University (No. M0397).

## Conflict of Interest

The authors declare that the research was conducted in the absence of any commercial or financial relationships that could be construed as a potential confliict of interest.

## Abbreviations

BC, Breast cancer; NC, Normal control; ER, Estrogen receptor; PR, Progesterone receptor; HER2, Human epidermal growth factor receptor 2; TNBC, Triple negative breast cancer; BRCA, Breast invasive carcinoma; IDBC, Invasive ductal breast carcinoma; ILBC, Invasive lobular breast carcinoma; LIHC, Liver hepatocellular carcinoma; CHOL, Cholangio carcinoma; ACC, Adrenocortical carcinoma; BLCA, Bladder Urothelial carcinoma; CESC, Cervical squamous cell carcinoma and endocervical adenocarcinoma; COAD, Colon adenocarcinoma; DLBC, Lymphoid Neoplasm Diffuse Large B-cell Lymphoma; ESCA, Esophageal carcinoma; GBM, Glioblastoma multiforme; HNSC, Head and Neck squamous cell carcinoma; KIRC, Kidney renal clear cell carcinoma; LAML, Acute Myeloid Leukemia; LGG, Brain Lower Grade Glioma; LUAD, Lung adenocarcinoma; LUSC, Lung squamous cell carcinoma; OV, Ovarian serous cystadenocarcinoma; PAAD, Pancreatic adenocarcinoma; PRAD, Prostate adenocarcinoma; READ, Rectum adenocarcinoma; STAD, Stomach adenocarcinoma; THCA, Thyroid carcinoma; THYM: Thymoma.
