# Corrigendum: Downregulation of GLYAT Facilitates Tumor Growth and Metastasis and Poor Clinical Outcomes Through the PI3K/AKT/Snail Pathway in Human Breast Cancer

**DOI:** 10.3389/fonc.2022.793448

**Published:** 2022-04-06

**Authors:** Xin Tian, Lina Wu, Min Jiang, Zhenyong Zhang, Rong Wu, Jianing Miao, Caigang Liu, Song Gao

**Affiliations:** ^1^ Department of Oncology, Shengjing Hospital of China Medical University, Shenyang, China; ^2^ Key Laboratory of Shengjing Hospital, China Medical University, Shenyang, China

**Keywords:** GLYAT, breast cancer, EMT, PI3K/AKT, clinicopathological features, prognosis

In the original article, there was a mistake in the legend for **Figure 2** and **Figure 3** as published. The scale bars of **Figures 2E, F, G**, **3A, B** were inaccurately described. The correct legends appear below.

**Figure 2:**
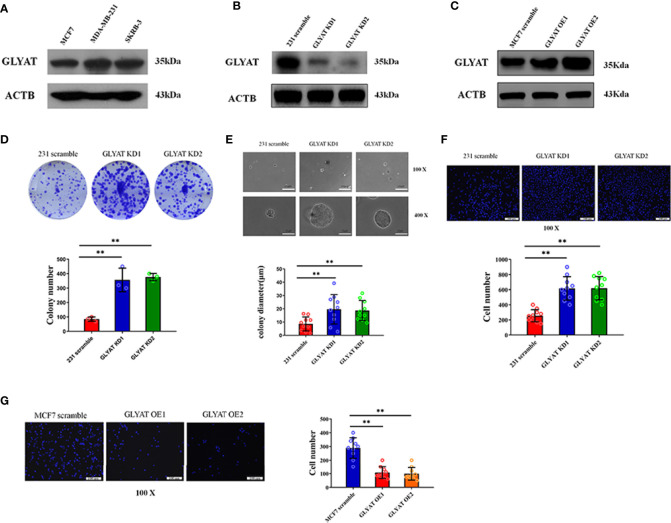
GLYAT suppresses BC cell proliferation and metastasis. **(A)** GLYAT protein level was assessed in different BC cell lines. **(B)** MDA-MB-231 cells were transfected with the GLYAT KD plasmid and a scrambled plasmid as control. The suppression of GLYAT in MDA-MB-231 cells was confirmed at the protein level. **(C)** MCF-7 cells were transfected with the GLYAT OE plasmid and a scrambled plasmid as control. The overexpression of GLYAT in MCF-7 cells was confirmed at the protein level. **(D, E)** GLYAT KD in MDA-MB-231 cells significantly increased colony numbers and size compared with the scramble cells. **(F)** The Transwell assay revealed that the migration ability of MDA-MB-231 cells was significantly increased following transfection with GLYAT KD1 and KD2 compared with the scramble control. **(G)** The Transwell assay revealed that the migratory ability was significantly decreased in GLYAT OE MCF-7 cells compared with scramble control. **p < 0.01. Scale bars for **(E)** are 100 μm and 25 μm, and for **(F, G)** are 100 μm.

**Figure 3:**
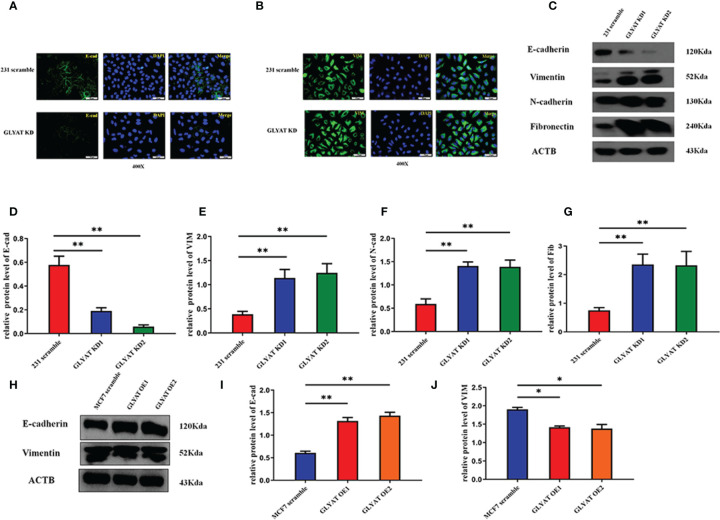
GLYAT suppresses EMT phenotype in BC cells. **(A, B)** The immunofluorescence assay showed that E-cadherin expression was decreased and vimentin expression was increased after the treatment of GLYAT KD. **(C–G)** Protein levels of E-cadherin was reduced, whereas the levels of vimentin, N-cadherin, and fibronectin were increased in MDA-MB-231 cells following GLYAT inhibition. **(H–J)** Protein levels of E-cadherin were increased whereas the expression of vimentin was reduced in GLYAT OE MCF-7 cells. *p < 0.05, **p < 0.01. Scale bars are 25 μm.

In the original article, there was a mistake in **Figure 2G** as published. We found that OE2 in **Figure 2G** was improperly placed during the figure editing process. The corrected **Figure 2** appears below.

In the original article, there was a mistake in **Figure 5I** as published. DAPI of MCF7 scramble p-AKT was wrongly placed. The corrected **Figure 5** appears below.

**Figure 5:**
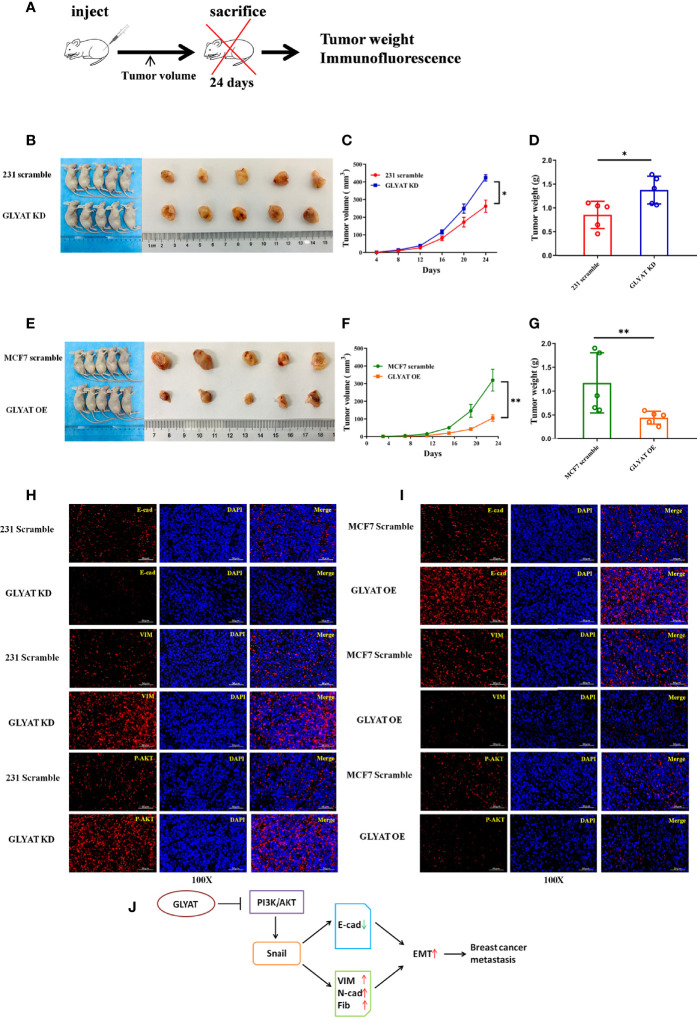
GLYAT suppresses breast cancer proliferation, metastasis, and EMT *in vivo*. **(A)** Schematic diagram of the metastasis model in mice. **(B–D)** The mice injected with stable GLYAT KD MDA-MB-231 cells had markedly bigger and heavier tumors. **(E–G)** The mice injected with stable GLYAT OE MCF-7 cells had markedly smaller and lighter tumors. **(H)** The immunofluorescence assay of serial sections of mouse tumor tissues revealed that GLYAT KD MDA-MB-231 cells significantly decreased the expression of E-cadherin whereas increased the expression of vimentin and p-AKT. **(I)** The immunofluorescence assay of serial sections of mouse tumor tissues revealed that, in GLYAT OE MCF-7 cells, the expression of E-cadherin was increased whereas vimentin and p-AKT were decreased. **(J)** The schematic diagram of the role of GLYAT in BC. *p < 0.05, **p < 0.01. Scale bars are 50 mm.

In the original article, there was a mistake in **Table 2** as published. In the process of statistical analysis, the statistical module of SPSS was wrongly selected. The corrected **Table 2** appears below.

**Table 2:** Univariate and multivariate analyses of clinicopathological risk factors for disease-free survival among breast cancer patients.

Variables	DFS			
	Univariate analysis		Multivariate analysis	
	HR (95% CI)	P value	HR (95% CI)	P value
Age	1.073 (0.751-1.533)	0.699	1.261 (0.847-1.878)	0.254
Menopausal status	0.603 (0.423-0.860)	0.005	0.123 (0.025-0.615)	0.011
T stage	1.117 (0.771-1.619)	0.557	1.142 (0.674-1.934)	0.621
N stage	0.974 (0.669-1.417)	0.890	0.951 (0.525-1.723)	0.869
TNM stage	0.745 (0.472-1.175)	0.206	0.806 (0.466-1.395)	0.442
Pathological type	0.864 (0.602-1.239)	0.427	0.953 (0.551-1.650)	0.864
Histological grade	0.911 (0.588-1.412)	0.677	0.837 (0.473-1.480)	0.540
ER status	0.712 (0.472-1.077)	0.107	0.812 (0.488-1.352)	0.424
PR status	0.730 (0.491-1.086)	0.120	0.759 (0.468-1.231)	0.264
HER-2 status	1.593(0.912-2.781)	0.102	1.727 (0.961-3.102)	0.068
Ki-67 status	0.871 (0.585-1.295)	0.495	0.797 (0.497-1.279)	0.347
Molecular subtype	0.802 (0.380-1.695)	0.564	0.932 (0.431-2.016)	0.858
GLYAT status	1.565 (1.098-2.232)	0.013	0.145 (0.031-0.690)	0.015

In the original article, there was an error in the results of multivariate analysis as the statistical module of SPSS was wrongly selected. A correction has been made to **Results**, *“Lower GLYAT Expression Is Correlated With Poorer Prognosis and Malignant Clinicopathological Features in Human Breast Cancer Tissues”*, paragraph 3:

“BC patients were analyzed for their GLYAT status and prognosis *via* Kaplan-Meier survival analysis as well as log-rank tests after being followed up for an average of 61.75 months (range, 9–77 months). We found that those with decreased GLYAT levels experienced poorer disease-free survival (DFS) (**Figure 6D**; P=0.012). GLYAT expression was a significant indicator of DFS rates in breast cancer patients based on univariate Cox regression analysis, demonstrating a hazard ratio (HR) of 1.565 [P<0.05; 95% confidence interval (CI) 1.098-2.232] (**Table 2**). Further multivariate analysis revealed that GLYAT expression was significantly related to DFS (HR, 0.145; 95% CI, 0.031-0.690; P=0.015) (**Table 2**) and represented an independent risk factor of prognosis for BC”.

After rediscussing the author contributions, all the authors agree to change the former co-responding author to co-author. The corrected **Author Contributions** Statement appears below.

The authors apologize for these errors and state that these do not change the scientific conclusions of the article in any way. The original article has been updated.

## Author Contributions

SG conceived and designed the experiments as well as contributed to the writing of the manuscript. XT, NW, MJ, YZ, NM, and RW performed the experiments. GL provided the tumor tissue microarray and brief guidance about conception at the beginning of the study. XT performed clinical analysis and helped with drawing the figures. SG revised the paper. All authors contributed to the article and approved the submitted version.

## Publisher’s Note

All claims expressed in this article are solely those of the authors and do not necessarily represent those of their affiliated organizations, or those of the publisher, the editors and the reviewers. Any product that may be evaluated in this article, or claim that may be made by its manufacturer, is not guaranteed or endorsed by the publisher.

