# Supplementary material for: Downregulation of GLYAT Facilitates Tumor Growth and Metastasis and Poor Clinical Outcomes Through the PI3K/AKT/Snail Pathway in Human Breast Cancer
Source: Front Oncol. 2021 Apr 22;11:641399. doi: 10.3389/fonc.2021.641399 (PMC8100313; doi:10.3389/fonc.2021.641399)
Supplement: Supplementary file 1 [file Image_1.pdf]

A

B

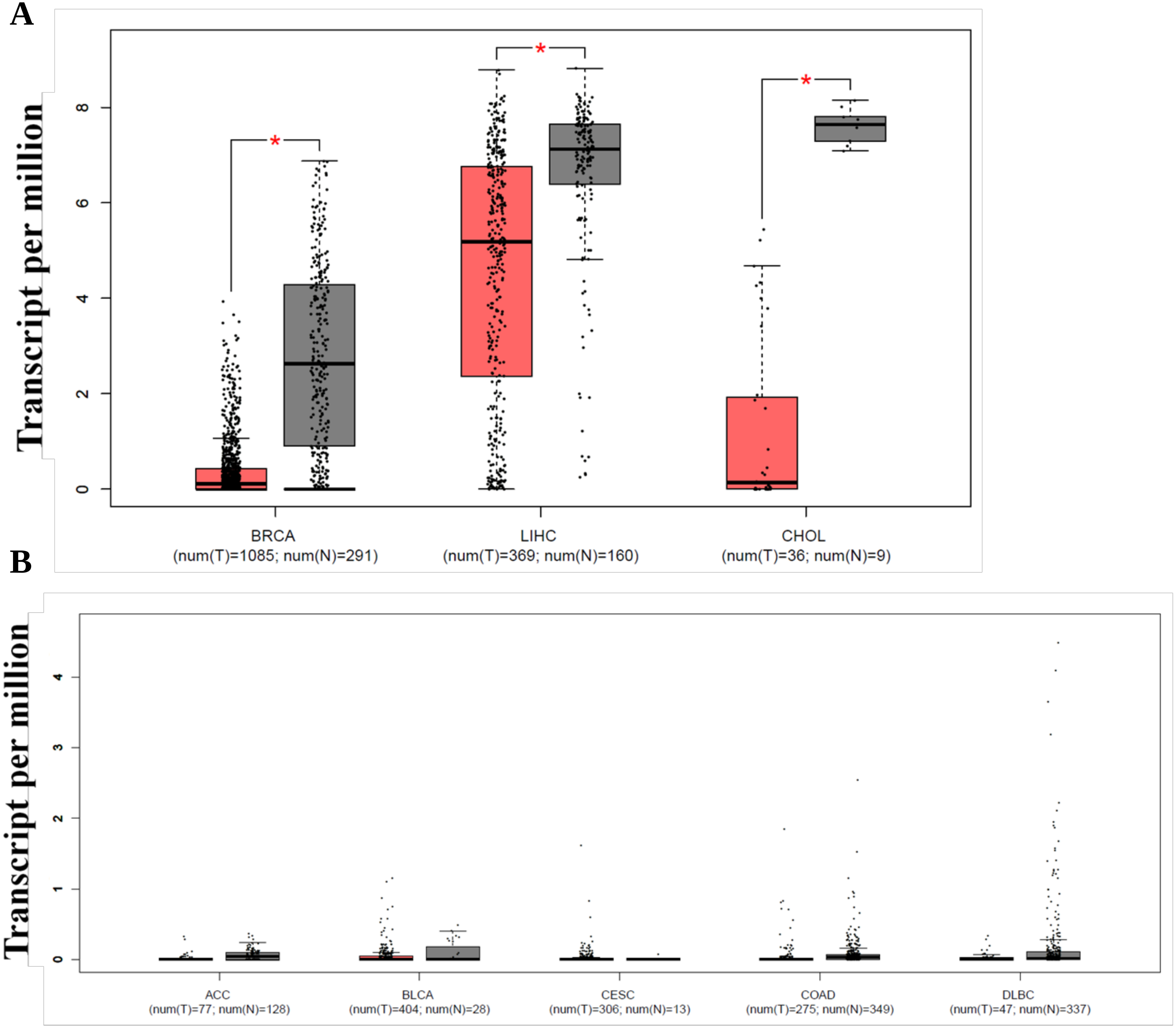
Transcript per million
0
2
4
6
8
BRCA
(num(T)=1085; num(N)=291)
LIHC
(num(T)=369; num(N)=160)
CHOL
(num(T)=36; num(N)=9)
Transcript per million
0
1
2
3
4
ACC
(num(T)=77; num(N)=128)
BLCA
(num(T)=404; num(N)=28)
CESC
(num(T)=306; num(N)=13)
COAD
(num(T)=275; num(N)=349)
DLBC
(num(T)=47; num(N)=337)

Fig 1

C

D

E

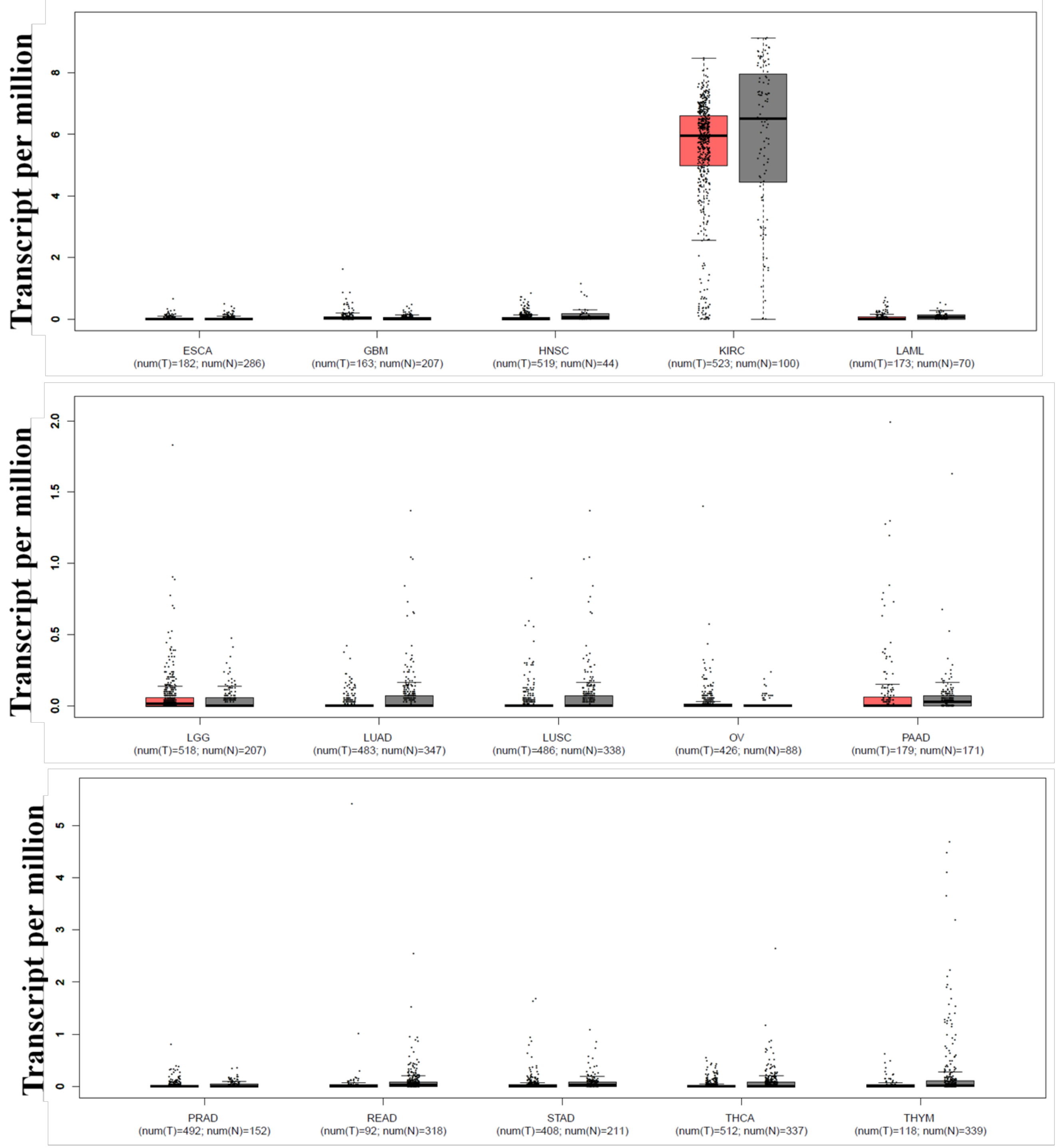

Transcript per million
ESCA
(num(T)=182; num(N)=286)
GBM
(num(T)=163; num(N)=207)
HNSC
(num(T)=519; num(N)=44)
KIRC
(num(T)=523; num(N)=100)
LAML
(num(T)=173; num(N)=70)
LGG
(num(T)=518; num(N)=207)
LUAD
(num(T)=483; num(N)=347)
LUSC
(num(T)=486; num(N)=338)
OV
(num(T)=426; num(N)=88)
PAAD
(num(T)=179; num(N)=171)
PRAD
(num(T)=492; num(N)=152)
READ
(num(T)=92; num(N)=318)
STAD
(num(T)=408; num(N)=211)
THCA
(num(T)=512; num(N)=337)
THYM
(num(T)=118; num(N)=339)

Fig 1

- **Figure 1. Box plot analysis showed the expression level of GLYAT in different tumor tissues. (A)** The GLYAT mRNA levels was decreased in BRCA, LIHC, CHOL. **(B)** There was no difference of the GLYAT mRNA levels in ACC, BLCA, CESC, COAD, DLBC and normal tissues. **(C)** The GLYAT mRNA levels was decreased in KIRC, but there was no difference in ESCA, GBM, HNSC, LAML and normal tissues. **(D)** The GLYAT mRNA levels was decreased in LGG and PAAD, but there was no difference of the GLYAT mRNA levels in LUAD, LUSC, OV and normal tissues. **(E)** There was no difference of the GLYAT mRNA levels in PRAD, READ, STAD, THCA, THYM and normal tissues.*p<0.05, **p<0.01.
