# Supplementary material for: Downregulation of GLYAT Facilitates Tumor Growth and Metastasis and Poor Clinical Outcomes Through the PI3K/AKT/Snail Pathway in Human Breast Cancer
Source: Front Oncol. 2021 Apr 22;11:641399. doi: 10.3389/fonc.2021.641399 (PMC8100313; doi:10.3389/fonc.2021.641399)
Supplement: Supplementary file 2 [file Table_1.docx]

Supplementary Material

**Supplementary Table 1** The sequence of pGPU6/mCherry/Puro-shRNA-GLYAT and pOGP-T2A-CKNeo-GLYAT-over-expression.

| pGPU6/mCherry/Puro-shRNA-GLYAT1 | Target sequencing：GGAGCAGGATATGACAGATGA |
| --- | --- |
| pGPU6/mCherry/Puro-shRNA-GLYAT2 | Target sequencing：GCAAACCCAAGGCCATCAACC   \|  \| \| --- \| |
| pOGP-T2A-CKNeo-GLYAT-over-expression | Target sequencing：CT ACC GGA CTC AGA TCT CGA GCT CAA GCT TATGATGTTACCATTGCAAGGTGCCCAGATGCTGCAGATGCTGGAGAAATCCTTGAGGAAGAGCCTCCCAGCATCCTTAAAGGTTTATGGAACTGTCTTTCACATAAACCATGGAAATCCATTCAATCTGAAGGCTGTGGTGGACAAGTGGCCTGATTTTAATACAGTGGTTGTCTGCCCTCAGGAGCAGGATATGACAGATGACCTTGATCACTATACCAATACTTACCAAATCTACTCCAAAGATCCCCAAAACTGTCAGGAATTCCTTGGATCACCAGAACTCATCAACTGGAAACAGCATTTACAGATTCAAAGTTCACAGCCTAGCCTGAATGAGGCTATACAAAATCTTGCAGCCATTAAGTCCTTCAAAGTCAAACAAACACAACGCATTCTCTATATGGCAGCTGAAACAGCCAAGGAACTGACTCCTTTCCTGCTGAAATCAAAGATTTTATCTCCCAATGGTGGCAAACCCAAGGCCATCAACCAAGAGATGTTTAAACTCTCATCCATGGATGTTACCCATGCTCACTTGGTGAATAAATTCTGGCATTTTGGTGGTAATGAGAGGAGCCAGAGATTCATTGAGCGCTGCATTCAGACCTTTCCCACCTGCTGTCTCCTGGGGCCTGAGGGGACCCCTGTGTGCTGGGATCTAATGGACCAGACTGGAGAGATGAGAATGGCAGGCACCTTGCCGGAATACCGGCTCCATGGCCTTGTGACGTATGTCATCTATTCCCACGCCCAGAAATTGGGCAAACTTGGGTTTCCTGTCTATTCTCATGTAGACTACAGCAATGAAGCTATGCAAAAAATGAGTTACACACTGCAACATGTTCCCATTCCCAGAAGCTGGAACCAGTGGAACTGTGTACCTCTG GTC GAC GGT ACC GAG GGC AGA GGA AGT CTT |
